# The Systematic Impact of Personal Characteristics on Entrepreneurial Intentions of Engineering Students

**DOI:** 10.3389/fpsyg.2020.01072

**Published:** 2020-06-03

**Authors:** Hongyi Sun, Wenbin Ni, Pei-Lee Teh, Carol Lo

**Affiliations:** ^1^Department of Systems Engineering and Engineering Management, City University of Hong Kong, Hong Kong, China; ^2^School of Business Administration, Zhejiang University of Finance and Economics, Hangzhou, China; ^3^School of Business, Monash University Malaysia, Selangor Darul Ehsan, Malaysia; ^4^Occupational Health and Safety Council of Hong Kong, Hong Kong, China

**Keywords:** personal characteristics, entrepreneurial intention, entrepreneurship education, need for achievement, risk-taking propensity, locus of control, creativity

## Abstract

The impact of personal characteristics on entrepreneurial intention is a classic topic in the field of entrepreneurship research. Previous research mostly used simple linear models, leading to a gap in the study on the interrelationship among personal characteristics and their systematic influence on entrepreneurial intention. This study investigates the interrelationship among the four specific entrepreneurial characteristics (i.e., need for achievement, locus of control, risk-taking propensity, and creativity) and their systematic influence on the entrepreneurial intention of engineering students. The research data is from 210 engineering students via a survey. Logistic regression and path analysis were used for data analysis. The findings suggest that creativity and risk-taking directly influence entrepreneurial intention while the need for achievement and the locus of control influence it indirectly. Implications for entrepreneurship education are finally discussed.

## Introduction

The impact of personal characteristics on entrepreneurial intention is a classic topic in the field of entrepreneurship research. Tremendous studies applied simple correlation, *t*-test, ANOVA, or linear regression to investigate the subject. However, the results are so inconsistent that the research nearly comes to an end. Coming into the 1980s and 1990s, scholars started to suspect the relationship between the two variables (Gartner, 1988, 1990; Behave, 1994). There are even references saying that no further research on personal characteristics is needed (Gartner, 1988, 1990). This background triggers the following research questions:

Do the findings on the influence of personal characteristics on entrepreneurial intention that insisted for several decades have no more meaning?
Do the inconsistent results of the effect of characteristics on intention thoroughly cast down the relationship between the two variables?
Do the behavioral models explain entrepreneurial intention perfectly?
Shall we continue to research the impact of personal characteristics in the period of entrepreneurial education?

There have been many studies on student entrepreneurial intention (Tkachev and Kolvereid, 1999; Lüthje and Franke, 2003; Wilson et al., 2007; Sun et al., 2017). However, İrengün and Arıkboğa (2015) suggest that education and training should center itself much more in changing personal attitudes than in knowledge. Personality is one of the factors that influence people’s attitudes. Frank et al. (2007) conducted a comprehensive study of different groups of people (e.g., secondary student students, university students, potential business founders, and successful business founders) and found different results for these groups using regression analysis. For example, for secondary school students, NACH and CA are significant entrepreneurial characteristics, while for university students, CA is not significant. For potential business founders and successful business founders, only RT significantly influences the entrepreneurial intention.

Therefore, it is timely research to re-consider the impact of personality on entrepreneurial intention for the following reasons.

Firstly, entrepreneurial individuals are the central part of entrepreneurship. Without the person, the action will never take place. However, not all people will become entrepreneurs even when circumstances are comparable. There must be some types of individual predisposition toward entrepreneurship, such as personal characteristics (Stewart, 1996). Therefore, personal characteristics are relevant for explaining the disposition of entrepreneurs to act entrepreneurially and why entrepreneurial behaviors differ under similar situations. Recent years see a revival of personality research in many areas such as performance, leadership, industrial, and organizational psychology (Rauch and Frese, 2000; Judge et al., 2002). Entrepreneurship is surely an active participant in this revival stream (Rauch and Frese, 2007a, b). Meta-analytic evidence (Collins et al., 2004; Stewart and Roth, 2004; Rauch and Frese, 2007b) suggests that personality does influence entrepreneurial intention and there has been a call to action for psychology for entrepreneurship research and practice (Hisrich et al., 2007), especially in entrepreneurial education (Hansemark, 1998; Frank et al., 2005; Hisrich et al., 2007) that aims at fostering entrepreneurial spirits and intentions.

Secondly, though the number of studies is huge, scholars have found incongruous results on the impact of personal characteristics on entrepreneurial intention during past decades. For example, some researchers reported a significant influence of the need for achievement (Howell and Higgins, 1990; Langan-Fox and Roth, 1995), while some did not (Bonnett and Furnham, 1991; Cromie et al., 1992; Ho and Koh, 1992; Koh, 1996). Incongruous results were also found for other characteristics such as locus of control, risk-taking propensity, and creativity. It is perhaps a methodological issue. Majority of the studies focused on the simple correlation of the characteristics and intention or comparison of different groups (e.g., entrepreneurs and non-entrepreneurs) (Robinson et al., 1991a, b; Cromie and O’Donoghue, 1992; Cromie et al., 1992; Ho and Koh, 1992; Carland et al., 1995; Green et al., 1996; Koh, 1996; Gurol and Atsan, 2006). Very few studies emphasized the interrelationships among the characteristics (Stewart, 1996). These types of research did not consider causal relationships and the issue of collinearity. Therefore some researchers claimed that the effect of personal characteristics on entrepreneurship had not been fully investigated (Johnson, 2003; Tett et al., 2003). Could there be significant interrelationship among the characteristics beyond their direct effect on intention? If the personal characteristics are measuring different perspectives of an individual, they are theoretically related to one another. This leads to another question: Is it possible that the relationships of the characteristics suppress their significant effect on intention? The influence of some characteristics on entrepreneurial intention is likely through the effect of others. This may help to illuminate the systematic influence of the characteristics and contribute to a better understanding of the impact. We clarify that the present study is not opposed to the behavioral approaches to entrepreneurship. Since neither personality nor behavioral approach fully explains the phenomenon, a comprehensive entrepreneurship model must consider a set of factors including background factors, personalities (broad and specific), attitudes, situational and contextual factors, and other factors that may affect the entrepreneurial intention, process, and performance. This study stresses the significance of personality, which is an inherent part of entrepreneurship study (Cromie et al., 1992; Stewart, 1996; Rauch and Frese, 2007a).

This study aims to investigate the empirical relationship between personal characteristics and entrepreneurial intention of engineering students. It focuses on two types of variables: entrepreneurial characteristics and intention. This study will contribute to (1) short-listing the most frequently cited personal characteristics that are pertinent to entrepreneurial intention, (2) exploring the interrelationship among these personal characteristics, (3) identifying the systematic impact of these personal characteristics on entrepreneurial intention, and (4) offering advice for designing entrepreneurship education programs in line with personal characteristics.

## Literature Review and Hypotheses

### Entrepreneurial Intention (EINT) and the Theory of Planned Behavior

Psychologists have claimed that an assessment of current intentions is the most obvious way of predicting the behavior itself (Ajzen, 1991). In various situations, intentions are the most effective predictor of behaviors such as job search activities and career choice (Kolvereid, 1996). The underlying assumption is that behaviors can be planned and under volitional control. That means a person can decide to do or not to do at all. This is called the Theory of Planned Behavior (Ajzen and Fishbein, 1980) that has been applied in many intention studies. The behaviors not planned or not under volitional control will not necessarily be directly determined by intentions and may thus result in a weak relation between intentions and behavior. Entrepreneurship is obviously planned. Entrepreneurs can decide to be involved in business creation or not. So it can be best predicted by the intention of the persons (Bird, 1988; Krueger and Carsrud, 1993). Many studies on entrepreneurship investigate entrepreneurial intention (Krueger et al., 2000; Carr and Sequeira, 2007), especially those on students (Davidsson, 1995; Autio et al., 1997; Fayolle et al., 2006a, b; Sun et al., 2017). In this study, entrepreneurial intention is defined as one’s judgments and attitude toward the likelihood of developing one’s venture and business (Grant, 1996). Before starting a new business, intentions to turn an idea into an actual business venture have been planned. The works by Bird (1988) and Krueger and Brazeal (1994) have shown that entrepreneurial behavior is the result of entrepreneurial intention. Studying intention gives us valuable insights into new venture initiation. For most students who are still on campus, the chance to start a new business is relatively low. It is difficult to collect data about entrepreneurial actions. So, the entrepreneurial intention is the most proper dependent variable if students are the subject of study.

### Personal Characteristics and the Trait Theory of Entrepreneurship

According to the trait theory of entrepreneurship, people who have entrepreneurial characteristics tend to have higher intentions to be involved in entrepreneurial activities (Caird, 1991; Cromie and O’Donoghue, 1992; Gurol and Atsan, 2006). The relationship will be elaborated in the following hypotheses.

#### Relationship Between Personal Characteristics and Entrepreneurial Intention (EINT)

Personal characteristics are significant factors in an entrepreneurship model (Mueller and Thomas, 2000). They are found associated with entrepreneurial motivation and intentions (Grant, 1996). Characteristics that predispose an individual to entrepreneurial intentions are called entrepreneurial characteristics. From an extensive review of nearly 80 publications on psychological entrepreneurship, we identified four most frequently cited entrepreneurial characteristics, namely, need for achievement (NACH, which has been cited 42 times), risk-taking propensity (RT, 36 times), locus of control (LOC, 33 times), and creativity (CA, 30 times). Others were cited only a few times. The four characteristics are also considered as the specific personalities which are proximal to entrepreneurship (Johnson, 2003; Rauch and Frese, 2007a). We extracted 21 most recent empirical studies covering one or all of the four characteristics and summarized them in Table 1.

**TABLE 1:** Empirical studies on entrepreneurship testing the personal characteristics.

		**NACH**	**RT**	**LOC**	**CA**	**Sample**	**Analysis method**
1	Howell and Higgins (1990)	v+	v+		v+	25 pairs of champions and no-champions of technological creativity	*t*-test, MANOVA, Pearson correlation, Regression analysis
2	Bonnett and Furnham (1991)	v		v+		190 secondary school and college students	ANOVA
3	Caird (1991)	v+	v+	v+	v+	262 business owners-managers and other occupation groups: teachers, nurses, civil servants, clerical trainees and lectures and trainers.	*t*-test
4	Robinson et al. (1991a)	v+		v+	v+	Totally 189 subjects: 49 businessmen, 22 students started own businesses, 50 white-collar non-managers, 68 psychology students	MANOVA, ANOVA
5	Robinson et al. (1991b)	v+		v+	v+	54 entrepreneurs and 57 non-entrepreneurs (white-collar non-managers	MANOVA
6	Cromie et al. (1992)	v	v+	v	v+	194 managers	ANOVA
7	Cromie and O’Donoghue (1992)	v+	v+	v+	v+	194 managers, 73 entrepreneurs and 661 undergraduates	*t*-test
8	Ho and Koh (1992)	v	v+	v+	v+	158 B Acc. Graduates	*t*-test
9	Carland et al. (1995)		v+			Entrepreneurs (*n* = 114), small business owners (*n* = 347), managers (*n* = 387)	*t*-test, ANOVA
10	Langan-Fox and Roth (1995)	v+		v		Female entrepreneurs	Cluster analysis
11	Palich and Bagby (1995)		v			35 entrepreneurs, 57 non-entrepreneurs	ANOVA
12	Green et al. (1996)	v+		v+		99 junior and middle managers of state enterprises and 108 first-generation small business founders	*t*-test, correlation
13	Koh (1996)	v	v+	v	v+	54 MBA students	*t*-test and Logit analysis
14	Stewart (1996)	v+	v+		v+	767 owner-managers of small business and corporate managers.	Multinomial logit regression
15	Chen et al. (1998)		v+	v	v+	100 business founders and 58 non-founders	ANOVA, Logistic regression
16	Hansemark (1998)	v+		v+		experimental groups (*n* = 19) and two control groups (*n* = 50)	*t*-test
17	Stewart et al. (1999)	v+	v+		v+	Entrepreneurs (*n* = 101), small business owners (*n* = 324), managers (*n* = 342)	*t*-test, Logit regression
18	Entrialgo et al. (2000)	v+		v+		233 SME managers	Regression analysis, Spearman correlation
19	Stewart and Roth (2001)		v+			Entrepreneurs and managers	Meta-analysis
20	Gurol and Atsan (2006)	v+	v+	v+	v+	400 university students	*t*-test
21	Frank et al. (2007)	v+	v	v	v+	875 secondary school students	Regression analysis
		v+	v+	v+	v	837 university students	
		v	v+	v	v	1,169 potential business founders	
		v	v+	v	v	754 successful business founders	
	Count (v +)	14	15	11	13		

*v, tested but not significant; v+, tested and significant.*

Different results exist regarding the impacts of the four characteristics on entrepreneurial intention (EINT). Among the 21 studies, only three studies (Caird, 1991; Cromie and O’Donoghue, 1992; Gurol and Atsan, 2006) covered all four entrepreneurial characteristics and found the same results that all the four personal characteristics were significantly different between entrepreneurs (or entrepreneurially inclined persons) and non-entrepreneurs (or those who are non-entrepreneurially inclined) based on their *t*-test results. However, using the same data analysis method, Ho and Koh (1992) argued that NACH did not have a significant impact on entrepreneurial inclination. Considering only three of the characteristics (NACH, RT, and CA), Howell and Higgins (1990); Stewart (1996), and Stewart et al. (1999) used regression analysis or logit analysis/logistic regression analysis and found that all the three characteristics were significantly related to entrepreneurial intention. However, using logit analysis or ANOVA, Cromie et al. (1992) and Koh (1996) posited that among the four characteristics, only two of them, RT and CA, exerted significant influence on entrepreneurial intention. While Bonnett and Furnham (1991) considered the effect of NACH and LOC and reported that LOC was significantly related to entrepreneurial intention, Langan-Fox and Roth (1995) found the opposite results (i.e., LOC was not significant). On the other hand, Hansemark (1998) using *t*-test and Entrialgo et al. (2000) using regression contended that both NACH and LOC were significant.

Although all the above studies considered NACH, RT, LOC, and CA as the independent variables and EINT as the dependent, they had different results. Concerning these inconsistent results as well as the relatively simple analysis methods used, we see a need to study the four characteristics with more sophisticated statistical tools such as SEM path analysis, which can study the postulated causal relationship considering all variables involved (Kline, 1998). Therefore, this study will first adopt the same structure to investigate the direct relationship between the entrepreneurial characteristics and intention using SEM analysis and then go deeper to study the inter-relationships among the characteristics. In this sense, the first four hypotheses are related to the direct relationship of NACH, RT, LOC, and CA to EINT.

NACH is the impetus that drives a person to struggle for success and perfection (Sagie and Elizur, 1999). RT is the propensity for risk-taking as the perceived probability of receiving the rewards associated with success before the potential entrepreneur actually subjects himself/herself to the consequences associated with failure (Brockhaus, 1980). LOC refers to an individual’s perceptions about the main underlying causes of events in his/her life. Rotter (1966) reported that LOC could be seen as either internal or external. Internals have higher achievement motivation than externals because internals believe their behavior is guided by their personal decisions and efforts, whereas externals feel guided by fate, luck, or other external circumstances. CA relates to perceiving and acting in new and unique ways (Robinson et al., 1991b).

The four characteristics have been recognized as the core elements that influence an entrepreneur’s decision making and behaviors, and can be used to differentiate entrepreneurs from the general people. For example, it has been found that entrepreneurs who have high NACH are more desirable to be successful and are subsequently more probably to behave creatively and entrepreneurially (Langan-Fox and Roth, 1995; Koh, 1996). They have also significantly higher than non-founders on RT because they have risk preferences (Shaver and Scott, 1991; Stewart and Roth, 2001; Raab et al., 2005) and have an internal locus of control attributed to their high self-esteem or confidence to control their lives (Entrialgo et al., 2000; Utsch and Rauch, 2000). Further, entrepreneurs are significantly more creative than non-entrepreneurs as they keep searching for new opportunities and taking a creative attitude toward their businesses (Utsch and Rauch, 2000). Based on the above discussions, we developed a foundation for proposing the following hypotheses concerning the personal characteristics and entrepreneurial intention:

**H1:** Need for achievement positively relates to entrepreneurial intention.

**H2:** Risk-taking propensity positively relates to entrepreneurial intention.

**H3:** Locus of control positively relates to entrepreneurial intention.

**H4:** Creativity positively relates to entrepreneurial intention.

#### Relationships Among the Four Personal Characteristics

Beyond the direct relationships between entrepreneurial characteristics and intention, this study goes deeper to explore the hidden inter-relationship among them. Most of the studies on entrepreneurial characteristics merely concentrated on the simple relationship between the characteristics and intention, and seldom touched upon the inter-relationship among the characteristics. Although Stewart (1996) studied the interaction between RT and NACH and between RT and CA, the author did not indicate the direction of the relationships. As a result, the previous studies seem to leave a gap for exploring how the personal characteristics influence one another in the formation process of EINT. This is important because it can bring in-depth insights into how the personal characteristics contribute to EINT and hence helps to explain their weak relationship obtained in previous studies. These will derive implications for entrepreneurship education aimed at nurturing the entrepreneurial spirits and intentions of students. If entrepreneurial characteristics can be learned or changed through education (Timmons et al., 1985; Hood and Young, 1993; Hansemark, 1998), understanding the relations among the characteristics will help educators to design teaching activities that nurture entrepreneurial characteristics more effectively. Therefore, this study accounts for the inter-relationship among the entrepreneurial characteristics.

According to McClelland (1987), high need achievers demonstrate a higher performance in challenging tasks (RT), look for tasks involving personal responsibility and display high self-esteem and confidence (LOC), and are creative in the sense of looking for new and better ways to improve their performance (CA). Therefore, the four characteristics measure different perspectives of an entrepreneur and influence one another.

NACH is highly related to a strong task, goal orientation, and an obsession with a task to be done (McClelland, 1985). People with high NACH are desirable to assume personal responsibility for performing a task, incline to set difficult goals, and are more eager to receive feedback (Cherrington, 1991). Since higher confidence will result in desirable outcomes, a high level of NACH is assumed. Thus, those who have internal LOC are likely to have a high level of NACH (Spector, 1982; McClelland, 1985). This relation is explained by the theories of intrinsic motivation (Deci and Ryan, 1985; Amabile and Hennessey, 1992) that internal locus of causality will contribute to intrinsic motivation. NACH, with its properties of need for competence and self-determination, is considered as a conceptualization of intrinsic motivation (Deci and Ryan, 1985). In accordance with the theoretical consistency between LOC and NACH, some studies have indicated that internal LOC positively influences NACH (Yukl and Latham, 1978; Abdel-Halim, 1980).

Although conversely, achievement motivation may enhance internal control belief based on the high achievement motivation, entrepreneurs will insist on taking personal responsibility for their performance. Scholars generally appear convinced that LOC results in achievement motivation (Colin, 1998; Reeve, 2001). Believing in one’s active influence, internals have higher motivation to reach success. In contrast, externally controlled people may be more passive. If one believes that he/she is not able to control business outcomes, he/she has no reason to actively change one’s environment (Rauch and Frese, 2007a). Therefore, we propose the following hypothesis:

**H5:** Locus of control positively relates to the need for achievement.

Control beliefs are energized by a need for control over meaningful life and business situations. People with internal LOC believe in their abilities to control their actions. Thus they are more tolerant of the risks when starting a business (Hillson and Murray-Webster, 2007). Internal LOC presupposes personal responsibility that drives individuals to pursue a high achievement in challenging environments. Entrepreneurs are thought to perform best in situations where they have personal responsibility for venture outcomes (Petrakis, 2007). Moreover, entrepreneurs are also confident in their abilities and exhibit resilience in the face of setbacks and can start over again when disappointments happen. They will pursue value creativity and do things not generally done in the ordinary course, and typically considered as innovators as well (Schumpeter, 2000; Drucker, 2007). Thus, internal LOC beliefs drive individuals to take risks and perform in creative ways. The evidence supports this belief that individuals with greater internal beliefs are more creative, risk-taking, and more entrepreneurial (Miller et al., 1982; Caird, 1991; Cromie and O’Donoghue, 1992; Chen et al., 1998). Therefore, we proposed that:

**H6:** Locus of control positively relates to risk-taking propensity.

**H7:** Locus of control positively relates to creativity.

People with a high level of needs tend to prefer challenging tasks (McClelland, 1985). In Atkinson’s (1957) formulation of risk-taking, NACH and fear of failure (i.e., anxiety about failing) have been considered as the operative and competing motives. The role of achievement motive in risk-taking was further explored by McClelland (1985) in relation to entrepreneurship. The individual’s needs and motives have been found to influence moderately risky and challenging tasks. McClelland believes that persons with high NACH have moderate RT. According to Atkinson (1957), a subjective probability of success of 50 percent is moderate risk and would generate the most achievement motivation. Stewart (1996) tested the relationship between RT and NACH and found a significant relationship between these two variables. However, the author did not specify the direction of the relationship. Hillson and Murray-Webster (2007) have posited that NACH enhances RT. They claimed that the level of achievement motivation affects an individual’s attitude toward the outcomes of risky situations, and therefore, the individual’s attitude toward risk. They also argued that high achievers are individuals who have a stronger motive to achieve relative to the motive to avoid failure, while low achievers have a stronger motive to avoid failure relative to their motive to achieve.

CA is another factor that is significantly influenced by NACH (Howell and Higgins, 1990). McClelland (1985) reviewed two decades of research and found that achievement-motivated individuals tend to move risk-taking like moving to another place to start a business and be more creative. “This is why entrepreneurial groups high in NACH are so fixated on finding a short cut to the goal that they may not be too particular about the means they use to reach it” (McClelland, 1985, p. 250). Utsch and Rauch’s (2000) study also stressed the positive relationship between the two variables. Thus, those with higher achievement motivation tend to be more creative. So the following two hypotheses are proposed.

**H8:** Need for achievement positively relates to risk-taking propensity.

**H9:** Need for achievement positively relates to creativity.

Finally, entrepreneurs explicitly see new and unusual solutions to problems, and they are expected to exhibit a greater degree of risk-taking (Cromie et al., 1992; Gurol and Atsan, 2006; Hillson and Murray-Webster, 2007). A significant relation between RT and CA was identified by Stewart (1996). Goldsmith (1994) and Howell and Higgins (1990) indicated that individuals who are willing to take risks tend to be more creative. Based on the discussion above, we postulate that:

**H10:** Risk-taking positively relates to creativity.

### The Conceptual Model

**FIGURE 1:**
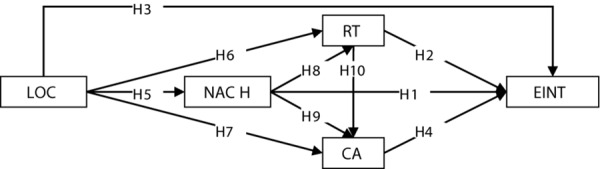
The conceptual model and hypotheses. LOC, locus of control; NACH, Need for achievement; RT, Risk-taking; CA, Creativity; EINT, Entrepreneurial intention.

The preceding discussion leads to the research model presented in Figure 1. In this model, we employ the concepts of both entrepreneurial characteristics and intention. As specified in the entrepreneurial literature, the four characteristics have a direct impact on entrepreneurial intention, respectively. Particularly, LOC predisposes individuals to risk-taking, achievement motivation, and creativity. NACH exerts a positive effect on both RT and CA. Lastly, RT is positively related to CA. This model has two basic features. Firstly, it comprises the characteristics of a typical trait model of entrepreneurship, demonstrating the specific impact of the personal characteristics on entrepreneurial intention. Secondly, it expands the inter-relations among the characteristics, providing a complete picture of how the entrepreneurial characteristics influence intention. That is, the model offers an in-depth and systematic approach to explain the influence of the characteristics on intention.

## Materials and Methods

### Survey Design

This study applied a cross-sectional survey design. This approach has been widely used in entrepreneurship studies (Krueger et al., 2000; Autio et al., 2001; Lüthje and Franke, 2003; Kristiansen and Indarti, 2004).

Questionnaires were sent to 300 students in engineering management courses (majored in manufacturing engineering and engineering management, and engineering management) in a university in Hong Kong. All questions were randomly printed without a logical sequence in versions that we distributed to the respondents. Also, there was no title for each section such that the students did not know the managerial content. Hence, the priming effects, item-context-induced mood states, and other biases related to the question context could be reduced (Podsakoff et al., 2003). Items measuring each of the constructs have been widely studied in entrepreneurship research (Rotter, 1966; Jackson, 1994) and have been considered valid and reliable with proven psychometric properties, which suggest that they are likely resistant to common method variance (Spector, 1987). Further, the items for each construct were different and no common items existed among the constructs. Hence it might help reduce confusion and ambiguity. Before the survey, a pilot study was also conducted among 10 engineering students to ensure clarity of all questions.

At the beginning of the survey, the respondents were told that the answers provided for selection did not mean the higher the better or the lower the better, and there was no right or wrong answers. All questions were anonymous. The survey was not a part of an exam or a form of evaluation and had nothing to do with the students’ performance. The students were told to answer the questions according to their true feelings about the questions, and the reliability of the completed questionnaires would be finally checked and that the improper ones would be screened out. These procedures help the respondents try to avoid providing similar answers to all questions, or providing answers on the basis to get social approval and acceptance, hence to reduce errors due to consistency motif and social desirability (Podsakoff et al., 2003).

Leniency bias (refers to the tendency for raters to rate their friends higher or better) was not relevant to our survey since the questions were not good or bad type, and they were about measuring the personalities of the students themselves. Implicit theory and illusion correlation (which refers to the rater’s assumption of co-occurrence and correlation of variables) was not concerned much in the study. The respondents were engineering students, not professional researchers in management. Further, they did not receive training in management correlation and management research. Moreover, the measure of personal characteristics (independent variables) and that of entrepreneurial intention (dependent variable) were different. The former was measure by 1–5 scale and the latter was categorical scale (Y/N). It seems difficult for those students to make consistency or implicit answers. Thus, the influence of implicit or explicit correlation was not problematic in this research.

Finally, this research does not aim at personality evaluation and is unlikely to seduce common method bias. Further, a set of procedural remedies have been applied to minimize the method biases. Therefore, the problem of common method bias may not seem to be a concern in this research. This was further confirmed by a Harmon one-factor test (Podsakoff and Organ, 1986), a widely used method to test the common method bias (Andersson and Bateman, 1997; Aulakh and Geneturk, 2000; Iverson and Maguire, 2000). If a single factor emerges from the factor analysis or one general factor that accounts for the majority of the covariance among the measure, common method bias is a problem. Results of the test suggested the presence of five factors, indicating that common method bias was not a pervasive problem in this study (Podsakoff and Organ, 1986; Andersson and Bateman, 1997; Aulakh and Geneturk, 2000) as well as the updated method (Tan et al., 2018; Loh et al., 2019).

### Profile of the Respondents

One hundred and forty-three usable questionnaires were returned with a 48 percent of usable response rate. Non-response bias was investigated by comparing the first 25 percent of the response with the last 25 percent of the response. No significant differences were found for several demographic characteristics such as age, education level, work experience, and average academic performance, suggesting that non-response bias was probably not an issue in the sample (Armstrong and Overton, 1977). One hundred and twenty-four of the samples were male students and 19 were female students. The students were in their bachelor’s (46.85%) or master’s (53.15%) study when they joined the survey. The respondents have an average age of 27. The average work experience was 4.7 years. Eighty-two respondents had entrepreneurial intention to establish their businesses soon, while 61 respondents lacked the intention to start their businesses.

### Measures

The questionnaires were developed based on five constructs: NACH, RT, LOC, CA, and EINT. Eight items measured each of the four characteristics. Respondents would indicate the extent to which they agreed or disagreed with each statement using a seven-point Likert scale, ranging from 1 (*strongly disagree*) to 7 (*strongly agree*). The higher scores they chose, the higher level of NACH, RT, internal LOC, and CA they perceived.

NACH was measured by a subset of items extracted from King (1985), while LOC was measured by Rotter’s (1966) scale used by Mueller and Thomas (2000). Items for RT and CA were adapted from the Jackson Personality Inventory (Jackson, 1994). The measurement was analyzed through factor analysis and reliability test. These data showed a subjects-to-variables ratio of 7.5:1, which is considered adequate for factor analysis (MacCallum et al., 1999; Hair et al., 2006). The data also showed an appropriate correlation matrix with substantial number of correlations greater than 0.30 (Flynn et al., 1994; Hair et al., 2006). Further, the Bartlett test of sphericity was significant and the Kaiser-Meyerr-Olkin measure of sampling adequacy was 0.807, which is far greater than the cut-off value of 0.6. In addition, measures of sampling adequacy (MSA) values were also well above the acceptable level of 0.5 (Coakes and Steed, 1999; Hair et al., 2006). Therefore, the data was appropriate to perform factor analysis. The remaining items of each of the entrepreneurial characteristics were exactly converged into their belonging factors, with loadings of 0.54 or above, which is practically significant (Hair et al., 2006). The total variance explained was 55 percent, which is higher than the acceptable level of 0.5 (Merenda, 1997). The values of Cronbach’s alpha were from 0.68 or above, indicating adequate internal reliability of the measurements (Nunnaly, 1978).

The measure of entrepreneurial intention was developed based on the item used in Krueger et al.’s (2000) study, in which a single-item variable was used. This study is interested in a general measure of intention to start up in the future rather than a specific measure (covering, such as specific kind of business to be created, or specific activities related to entrepreneurship to be performed). According to Nagy (2002) and Wanous et al. (1997), a single-item measure of a general construct is appropriate. Further single-item scales are less time-consuming and are not monotonous for the respondents to complete (Gardner et al., 1998), as many potential respondents are not willing to spend time to complete an extensive survey (Pomeroy et al., 2001).

Despite its inherent limitation, the single-item measure has been used in many different disciplines, including business (Nagy, 2002), education (Wanous and Hudy, 2001), and psychology (Killgore, 1999; Robins et al., 2001). These authors noted several advantages of single-item measures, such as increased face validity and flexibility, good reliability, and adequacy. In entrepreneurship, single-item scales measuring entrepreneurial intention have also been verified by researches. For example, Koh (1996); Krueger et al. (2000), and Peterman and Kennedy (2003) used a single item to measure of the entrepreneurial intention of respondents by asking if they have entrepreneurial intention. Similarly, in our study, the students were asked if they had intention to establish their own business in the future.

Demographic information was also collected to develop a profile of the sample and verify that respondents who had entrepreneurial intention and those who had not been homogeneous with respect to demographic factors. This helped ensure that such factors did not confound the results. For this purpose, questions on age, gender, education level, work experience, and academic performance were included in the questionnaire. The questionnaire is listed in Appendix.

### Data Analysis Methods

A set of statistical methods (using SPSS and Amos) was employed. The first step of the analysis involved a chi-square test, which was used to verify that the respondents who had entrepreneurial intention and those who had not in the sample were homogeneous with respect to their demographic characteristics. Secondly, *t*-tests were conducted to test if significant differences regarding the four characteristics existed between the two subgroups. Then binary logistic regression and SEM path analysis were used to evaluate the conceptual model and to test the 10 null hypotheses specified in the study. Binary logistic regression (logistic model or logit model) was employed because it is useful to describe the relationship between one or more factors and an outcome (which only takes two possible values: yes or no) (Agresti, 2002). Thus, it is suitable to analyze the direct effect of the four characteristics on entrepreneurial intention, which is a dichotomy in nature (i.e., H1–H4). The sample size of 143 of this study satisfies the requirement of logistic regression analysis, where the ratio of 20 observations for each predictor variable is suggested (Hair et al., 1998). In the logistic regression model, the intention was the dependent variable, and the four characteristics were independent.

The relationships among the characteristics (i.e., H5–H10) were tested by path analysis, a subset of Structural Equation Modeling (SEM). In the path model, LOC was exogenous variable, while the other three characteristics were endogenous variables. The SEM path analysis used in this study has two advantages: (1) It simultaneously tests all relationships within the model; and (2) It tests the goodness of fit for different nested models (Anderson and Gerbing, 1988). The relationship among the characteristics can be tested simultaneously with path analysis [using Amos 16.0 with maximum likelihood estimation (MLE)]. The sample size of this study just fulfilled the requirement that a sample size of 100–200 is recommended to have confidence in the goodness of fit test (Hoyle, 1995). In terms of model fit, the following indices were used: Chi-square (x^∧^2) statistics, Chi-square statistics divided by the degree of freedom (x^∧^2/df); Goodness-of-fit index (GFI), Adjusted goodness of fit index (AGFI), Comparative fit index (CFI), Normed fit index (NFI), Tucker-Lewis coefficient (TLI), and Root mean square error of approximation (RMSEA). As suggested in the literature (Kline, 1998), the following criteria of the indices were used to assess the model-fitting: x^∧^2 statistic is not significant (*p* > 0.05); x^∧^2/df ratio is recommended to be less than 3; the values of GFI, AGFI, CFI, NFI, and TLI are recommended to be greater than 0.90; and RMSEA is recommended to be up to 0.05, and acceptable up to 0.08.

## Analysis and Results

### Demographic Factors

The objective of this study is to investigate the systematic influence of personal characteristics on entrepreneurial intention of engineering students. Chi-square tests of independence were performed to investigate the impact of the demographic factors on the decision of the students on performing entrepreneurial acts to ensure factors such as demographic variables did not confound the results. Thus, the differences with respect to the age, gender, education level, work experience, and average academic performance between those who had entrepreneurial intention and those who had not were tested. The results are reported in Table 2. None of the demographic factors was significantly different between the two groups at the 0.05 significance level. Accordingly, these demographic factors had no significant impact on entrepreneurial intention. That is, the two groups of respondents could be considered homogeneous concerning their backgrounds. Given the results, it was possible to test if entrepreneurial intention was significantly associated with the four characteristics without the confounding effects of demographic variables.

**TABLE 2:** Effect of demographic factors.

**Demographic factors**	**Value**	**df**	**Asymp. Sig (2-sided)**
Age	0.410	3	0.938
Gender	0.303	1	0.582
Education Level	1.389	2	0.499
Work Experience	0.705	2	0.703
Average Academic Performance	1.365	2	0.505

The mean scores shown in Table 3 were consistent with expectations reflected in the literature review and indicated that those who with entrepreneurial intention had greater need for achievement and higher propensity to take risk, and were more internally controlled and creative. To investigate the differences statistically at the univariate level, *t*-tests were conducted (Table 3). At a 0.05 significance level, the results showed that the entrepreneurially intended had significantly higher NACH (*p* = 0.005) and propensity to take risk (*p* = 0.000), and were more internally controlled (*p* = 0.001) and creative (*p* = 0.000). These imply that the characteristics were related to entrepreneurial intention and provide evidence for further investigation of their systematic impact.

**TABLE 3:** Differences of the four characteristics between the two subgroups.

	**Entrepreneurial intention**						
	**Yes**		**No**		***t*-test**		
**Variables**	**Mean**	**Sd**	**Mean**	**Sd**	**n**	***t*-value**	**Sig. (2-tailed)**
NACH	5.45	1.11	5.04	1.29	112	−2.854	0.005
RT	4.86	1.16	3.74	1.30	141	−8.369	0.000
LOC	5.29	1.15	4.78	1.26	141	−3.473	0.001
CA	5.25	1.07	4.72	1.36	141	−4.083	0.000

### Tests of Hypotheses

#### Direct Effect of the Personal Characteristics on Entrepreneurial Intention (H1–H4)

Performing a logistic regression analysis to test H1–H4. Table 4 summarizes the results. The Hosmer-Lemeshow (H-L) Chi-square test for the logistic regression model yielded a *p*-value of 0.279, thus suggesting a good fit. The holdout accuracy rates of the logistic regression model are presented in the upper part of the table. When the four characteristics enter into the model equation simultaneously, the overall accuracy rate of the final model was good at 81.1 percent, which is much higher than blindly estimating the most frequent category (entrepreneurial intention) for all cases at 57.3 percent. This accuracy rate is acceptably high compared with the figure reported by Robinson et al. (1991b), 77 percent, using NACH, LOC, CA, and self-confidence to predict entrepreneurial intention. Therefore, the four characteristics respectively had a significant effect on EINT of the engineering students. The importance of specific parameters of the model is reflected in the lower part of Table 4. Using *p* = 0.05 as a cutoff criterion for not including variables in the equation, it appears that RT (**H2**) and CA (**H4**) are important predictors of entrepreneurial intention, while NACH (**H1**) and LOC (**H3**) did not appear to be important. Thus, H2 and H4 were supported while **H1** and **H3** were rejected.

**TABLE 4:** Direct relationship between the personal characters and intention (H1–H4)*.

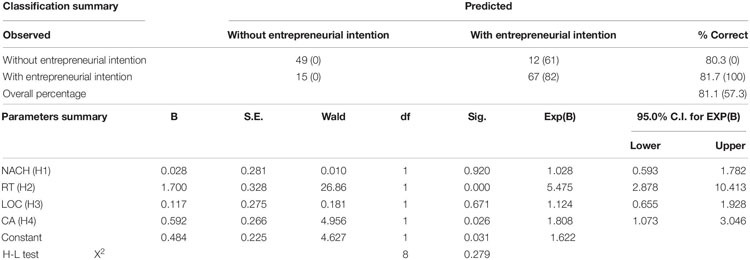

( ) only constant was included in the model. *Logistic regression models: variables entered NACH, RT, LOC, CA and the cut value was 0.500.

#### The Relations Among the Four Personal Characteristics (H5–H10)

H5 to H10 postulating the relationships among the four characteristics were tested with path analysis. Since the hypothesized path model was saturated, perfect model fit was obtained (i.e., GFI = NFI = CFI = 1.0), but the path coefficient from RT to CA was not significant (**H10**). This path was eliminated and the modified model was tested again. The results are shown in Table 5. As can be seen in the new model, all relationships hypothesized were significant (path LOC → NACH and NACH → CA were significant at *p* = 0.001; LOC → CA and LOC → RT at *p* = 0.05; NACH → RT was marginally significant at *p* = 0.1). A chi-square of 1.235 (*df* = 1; *p* = 0.267), x^∧^2/df ratio (1.235), other goodness-of-fit statistics greater than 0.9 (GFI = 0.996; AGFI = 0.957; CFI = 0.998; NFI = 0.989; TLI = 0.987) and REMSEA (0.041) indicated good model fit. In general, the model explained 34 percent of NACH, 14 percent of RT, and 22 percent of CA. Therefore, **H5, 6, 7, 8, and 9** were supported. LOC had a positive effect on NACH, RT, and CA, respectively; NACH was positively related to RT and CA. However, RT was failed to relate to CA. Thus, **H10** was not supported.

**TABLE 5:** Relationship among the four personal characteristics (H5–H10).

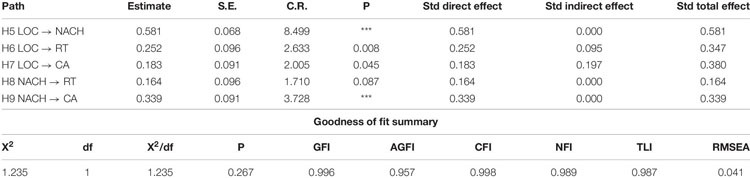

***p-value < 0.001.

**FIGURE 2:**
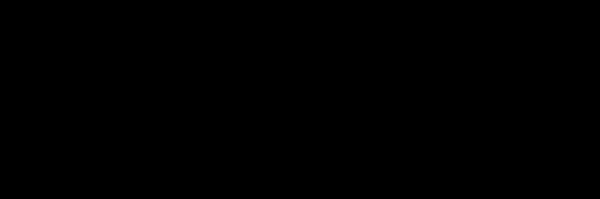
Influence of the personal characteristics on entrepreneurial intention. LOC, locus of control; NACH, Need for achievement; RT, Risk-taking; CA, Creativity; EINT, Entrepreneurial intention.

In sum, the test results (significant relationships) are summarized in Figure 2. It can be seen that the four characteristics influenced entrepreneurial intention in a holistic way. LOC positively affected the other three characteristics and NACH had a positive impact on RT and CA, which exerted a direct effect on the intention.

## Discussion and Implications

### Research Method Implications

This study investigated the systematic influence of the four specific entrepreneurial characteristics (NACH, RT, LOC, and CA) on EINT through examining their inter-relationships. Most of the existing studies on the relationship between personal characteristics and intention focused on examining the responses of entrepreneurs and non-entrepreneurs (Robinson et al., 1991b; Carland et al., 1995; Palich and Bagby, 1995; Chen et al., 1998), those who were entrepreneurial oriented/inclined and those who were not (Koh, 1996; Gurol and Atsan, 2006), business-owners/managers and other occupation groups (Caird, 1991), and students, entrepreneurs, and managers (Cromie and O’Donoghue, 1992). These results relied on the analysis of *t*-test, Pearson correlation, ANOVA, or simple linear model (Bonnett and Furnham, 1991; Cromie and O’Donoghue, 1992; Cromie et al., 1992; Ho and Koh, 1992; Green et al., 1996; Gurol and Atsan, 2006). It seems challenging to reveal the comprehensive influence of the personality characteristics on intention based on the simple correlations of the characteristics and the comparison results of different groups. Therefore, as highly emphasized in the review study of Dean et al. (2007), more sophisticated research designs are needed for hypothesis testing in the field of entrepreneurship. This study investigated the systematic effect of entrepreneurial characteristics on EINT using SEM path analysis, a sophisticated statistical method which is appropriate to understand the critical aspects of entrepreneurship (Brush et al., 2003; Dean et al., 2007).

### Theoretical Implications

Results of the current study (in Table 4) indicated that collectively, the four characteristics could distinguish those who had entrepreneurial intention from those who had not at an overall holdout accuracy rate of 81.1 percent, which could be considered adequate. The results showed that the engineering students who had a higher chance to take risk and were more creative tended to create their businesses. The findings supported the literature on trait model that people who possess the entrepreneurial characteristics tend to have higher intention to perform entrepreneurial acts and are consistent with the findings of Caird (1991); Cromie and O’Donoghue (1992), and Gurol and Atsan (2006). The findings were also in line with the logit analysis results of Koh (1996), where risk-taking and creativity were the predictors of entrepreneurial inclination.

Although NACH (**H1**) and internal LOC (**H3**) were not found to influence the students’ entrepreneurial intention directly, they had a significant indirect effect. For example, LOC affected ENIT through its effect on the other three characteristics (**H5–H7**), and NACH affected ENIT through RT and CA (**H8–H9**) (Table 5). That is, the students who had internal control beliefs would have higher achievement motivation, higher propensity to take risk, and be more creative. Further, those who had higher entrepreneurial motivation would have stronger desire to take risk and adopt creative ideas. The current results concurred with the previous findings that internal locus of control leads to high motivation (Colin, 1998; Reeve, 2001), thus predicts preference for risky and challenging tasks (Hillson and Murray-Webster, 2007) and turns individuals to be less conforming and do things not generally done in the ordinary course of business routine (Schumpeter, 2000; Drucker, 2007).

RT and CA (**H10**) has no significant relationship. This finding could be thought of as the consequence of the study environment focusing on learning of theories, which lacked actual practice. For engineering students, they had few opportunities to take risky business actions in their studies, particularly related to creating a new venture. Thus the students might not comprehend risk-taking in relation to entrepreneurship. In this case, CA and RT could be considered as two independent concepts for the students as their creative mind was not attributed to their RT. In short, in the context of engineering students, CA was not affected by RT.

Finally, the most important theoretical implication is about the adoption of the theoretical model in intention research. This research indicated that no matter if based on the trait theory or behavior theory, the model should include the systematic impact of personal characteristics in their models. In recent years, intention-based models, such as the theory of planned behavior (TPB) (Ajzen, 1991), Shapero’s entrepreneurial event model (Shapero and Sokol, 1982), and Bird’s intention model (Bird, 1988), have been used in the field of entrepreneurship to capture the link between an individual and his or her actions. Particularly, TPB has received more attention from scholars to explain the planned behavior of starting a business, which is best predicted by intentions (Krueger and Carsrud, 1993). These models take into account the impact of factors such as attitudes (Ajzen, 1991), social pressure (Shapero and Sokol, 1982; Ajzen, 1991), perceived behavioral control (Shapero and Sokol, 1982; Ajzen, 1991), and goals of starting a business (Bird, 1988), little concern about personal characteristics is considered in these models. According to Ajzen (2005), personality traits are one of the background factors that influences attitudes of the TPB model.

### Practical Implications for Entrepreneurship Education

The findings of the current study bear practical implications for entrepreneurship education that aims at enhancing entrepreneurial intention of engineering students. Researchers have suggested that entrepreneurs are not born; they are made (Gorman et al., 1997; Fiet, 2001a,b). The trait model can be applied to entrepreneurship education (Koh, 1996; Hansemark, 1998) and the psychological attributes have been recognized as important for entrepreneurial activity and economic growth (Singh, 1989; Shaver and Scott, 1991). Although personality characteristics are generally stable and not subject to cursory change, some researchers have provided evidence that some characteristics (specific personal characteristics to entrepreneurship such as NACH, RT, LOC, and CA) can be learned or enhanced through entrepreneurial training and education (McClelland and Winter, 1969; Miron and McClelland, 1979; Timmons et al., 1985; Hood and Young, 1993; Hansemark, 1998). Rotter (1966) recognized that internal LOC beliefs could be learned through training and education experiences. McClelland and Winter (1969) and Miron and McClelland (1979) showed that NACH could be developed, although the training programs were for under-achieving students, minorities, or in under-developed areas. On the other hand, Heckhausen and Krug (1982) found that NACH is learnable. Studying the effect of a formal entrepreneurship program (aimed at developing abilities, knowledge, skills, attitudes, and personal attributes which are important for entrepreneurial activities) on personal characteristics, Hansemark (1998) found that participation in an entrepreneurship education program would lead to a higher level of NACH and increase internal orientation of LOC. Although his study did not explicitly explain which elements of the program caused the results, it clearly indicated that personal characteristics, which are related to entrepreneurial action, could be developed and that this could be done through entrepreneurship education. Further, in Hood and Young’s (1993) research, the importance of “teaching” the personal characteristics such as RT and CA in entrepreneurship education to train successful entrepreneurs was also emphasized.

Working on these premises, the contents of entrepreneurship education should enhance or facilitate the development of entrepreneurial characteristics. Educators that seek ways in which to foster entrepreneurial intention of students need to recognize the holistic influences of these factors. This research provides a sequence in personal development via entrepreneurship education. The findings suggest that internal LOC is the elementary part of the influencing chain. It will facilitate the development of NACH, RT, and CA. Educators can develop LOC focusing on confidence building, commitment, and personal responsibility for the goals they set. The purpose is to help students to exhibit self-confidence in showing their abilities to perform tasks and insist on taking personal responsibilities for their performance. The next step is to develop students’ desire for achievement by practicing challenging tasks or games (from easy to difficult levels) or set different levels of goals of achievement during the entrepreneurship program/course (e.g., short-term and long-term realistic goals). According to Hansemark (1998), when developing internal LOC and NACH, the students must believe in their ability to bring about change and to control their own lives. Those who with more internal locus of control feel more confident and capable of performing entrepreneurial acts, thus facilitating their RT and CA, which will finally lead to the formation of entrepreneurial intention. Based on Hood and Young’s (1993) research, RT can be effectively trained by business experience, class discussion, discussion with business people/leaders, mentoring and seminars, while CA can be trained through class experiential exercises (including creativity games and exercises), discussion groups, case studies, mentoring, simulation, and game theory, which are effective pedagogical vehicles for inspiring students’ innovative thinking, changing their mentalities or learning new ones.

## Conclusion and Future Research

This study explores the systematic influence of personal characteristics (NACH, RT, LOC, and CA) on EINT by examining the inter-relationships among the personal characteristics. This is perhaps the first research to reveal how the characteristics, directly and indirectly, influence entrepreneurial intention. Sophisticated statistical methods (logistic regression and SEM path analysis) were used for data analysis. The results showed that RT and CA were the dominant direct predictors of EINT of the engineering students, while LOC and NACH had indirect influences. For example, LOC facilitated the other three characteristics, and NACH facilitated both RT and CA. The findings support the proposition that the four characteristics influence EINT holistically, instead of individually. This confirms the value of personal characteristics in the traditional trait model and provides new insights into understanding their impact on EINT. The contributions of this research include (1) filling in the literature gap on the interrelationship among personal characteristics; (2) filling in the literature gap on the holistic influence of personal characteristics on EINT; (3) removing the doubt on whether personal characteristics are worthwhile to be studied; (4) applying SEM path analysis to examine the interdependent effect of personal characteristic on EINT; and (5) exploring a few new areas for future research on personal characteristics as well as their combination with education and behavior models.

There are a few limitations in this study which may lead to future research. Firstly, this study focuses on engineering students in Hong Kong. Future research can be expanded to students of different disciplines, in different years of study, or from other countries. It can also be conducted in secondary students as researcher conducted (Paço et al., 2011) or extend the research from intention to activities (Raposo and Paço, 2011).

Secondly, this study is based on a cross-section survey. A longitudinal study in the future on entrepreneurship programs/courses could help us measure the effects of education on students’ personal characteristics and the effect of intentions on actual entrepreneurial behaviors. Thirdly, the sample size of this study fulfills the requirement of factor analysis, logistic regression analysis, and achieves the minimum requirement of SEM path analysis. Larger sample size is suggested to get more stable results (Hair et al., 1998). Fourthly, the model was tested separately by two different methods, logistic regression and SEM path analysis, that the general analysis of the entire model was hidden due to the dichotomous nature of the dependent variable. This research can be repeated by modifying the dependent variable into a numerical scale (such as a 5-point Likert scale). Hence, the whole model can be analyzed altogether by SEM path analysis and more statistically convincing results can be obtained.

Finally, we suggest that future models consider the systematic influence of the personal characteristics on EINT or on the intention models. Background factors and contextual factors may also be included to have a complete model. Since personal characteristics can be developed or enhanced through education, such integration is particularly important to develop an entrepreneurship education model that explains how education fosters the entrepreneurial intention of students through developing their entrepreneurial characteristics as well as acquiring relevant knowledge and skills. With its strong flux of renewed interest, entrepreneurial characteristics are set to be an even more important area for academic and professional research in the future rather than being a label of convenience.

## Data Availability Statement

The raw data supporting the conclusions of this article will be made available by the authors, without undue reservation, to any qualified researcher.

## Ethics Statement

Ethical review and approval was not required for the study on human participants in accordance with the local legislation and institutional requirements. Written informed consent from the participants was not required to participate in this study in accordance with the national legislation and the institutional requirements.

## Author Contributions

HS: writing. WN: conceptualization and writing. P-LT: writing – review and editing. CL: data collection and analysis.

## Conflict of Interest

The authors declare that the research was conducted in the absence of any commercial or financial relationships that could be construed as a potential conflict of interest.

## Appendix | The Questionnaire

Section 1: Your personal information (tick or circle only one choice).

a. Age: <25:___, 25–30:___, 31–35:___, >35:___.
b. Gender: Male: ___, Female: ___.
c. Education: High diploma: ___, Bachelor: ___, Master: ___, PhD: ___.
d. Years of working experiences: <5:___, 5–10:___, >10:___.
e. Your estimated average performance for all education: A:___, B:___, C:___.
f. Do you have the intention of establishing your own business in the future: Yes:___, No:___.

Section 2: Personal characteristics.

Strongly disagree = 1 2 3 4 5 6 7 = Strongly agree

**Table:**

**Alpha (loading)**	**Code**	**Questions**	**Measure scale**						
**0.77**	**NACH**								
(0.72)	NA1	My aim in life is to make a long record of successful achievements.	1	2	3	4	5	6	7
(0.65)	NA2	I like to do my best in whatever work I undertake.	1	2	3	4	5	6	7
(0.85)	NA3	I frequently desire to do something of great significance.	1	2	3	4	5	6	7
(0.79)	NA4	I often desire to be successful in doing something very significant.	1	2	3	4	5	6	7
(0.64)	NA5	For pleasure and happiness one must enrich the record of one’s achievements.	1	2	3	4	5	6	7
deleted	NA6	I have a great need for performance feedback.	1	2	3	4	5	6	7
deleted	NA7	I am happiest when I am successful in my work.	1	2	3	4	5	6	7
deleted	NA8	Achievements motivate me more than anything else.	1	2	3	4	5	6	7
**0.82**	**RT**								
deleted	RT1	I will sometimes stretch out on a limb to get the things I want.	1	2	3	4	5	6	7
deleted	RT2	I would enjoy bluffing my way into an exclusive club or private party.	1	2	3	4	5	6	7
(0.73)	RT3	If the possible award was very high, I would not be hesitating putting my money into a new business that could fail.	1	2	3	4	5	6	7
(0.78)	RT4	People have told me that I seem to enjoy taking chances.	1	2	3	4	5	6	7
(0.72)	RT5	The thought of investing in stock excites me.	1	2	3	4	5	6	7
(0.77)	RT6	I enjoy taking risks.	1	2	3	4	5	6	7
(0.74)	RT7	Taking risks does not bother me if the gains involved are high	1	2	3	4	5	6	7
(0.63)	RT8	I would enjoy the challenge of a project that could mean either a promotion or loss of a job	1	2	3	4	5	6	7
**0.69**	**LOC**								
(0.80)	LC1	Whether or not I get to be a leader depends mostly on my ability.	1	2	3	4	5	6	7
deleted	LC2	Whether or not I get into a car accident depends mostly on how good a driver I am.	1	2	3	4	5	6	7
deleted	LC3	When I make plans, I am almost certain to make them work.	1	2	3	4	5	6	7
deleted	LC4	How many friends I have depends on how nice a person I am.	1	2	3	4	5	6	7
deleted	LC5	I can pretty much determine what will happen in my life.	1	2	3	4	5	6	7
(0.54)	LC6	I am usually able to protect my personal interest.	1	2	3	4	5	6	7
(0.84)	LC7	When I get what I want, it is usually because I work hard for it.	1	2	3	4	5	6	7
(0.69)	LC8	My life is determined by my own action.	1	2	3	4	5	6	7
**0.68**	**CA**								
deleted	CA1	I prefer work that requires original thinking.	1	2	3	4	5	6	7
(0.73)	CA 2	I am always seeking new ways to look at things.	1	2	3	4	5	6	7
deleted	CA 3	Original ideas have occurred to me at almost any time of the day.	1	2	3	4	5	6	7
deleted	CA 4	I enjoy thinking of original plans on which to work.	1	2	3	4	5	6	7
(0.54)	CA 5	I often surprise people with my novel ideas.	1	2	3	4	5	6	7
deleted	CA 6	People often ask me for help in creative activities.	1	2	3	4	5	6	7
(0.77)	CA 7	I hope to develop new techniques in my field of work.	1	2	3	4	5	6	7
(0.77)	CA 8	I like to experiment with various ways of doing the same thing.	1	2	3	4	5	6	7
